# Common Genetic Factors and Pathways in Alzheimer’s Disease and Ischemic Stroke: Evidences from GWAS

**DOI:** 10.3390/genes14020353

**Published:** 2023-01-30

**Authors:** Wei Dong, Yue Huang

**Affiliations:** 1 China National Clinical Research Center for Neurological Diseases, Beijing Tiantan Hospital, Capital Medical University, Beijing 100070, China; 2 Department of Neurology, Beijing Tiantan Hospital, Capital Medical University, Beijing 100070, China; 3 Department of Pharmacology, School of Medical Sciences, Faculty of Medicine & Health, UNSW, Sydney, NSW 2052, Australia

**Keywords:** Alzheimer’s disease, ischemic stroke, genetics, molecular pathways

## Abstract

Alzheimer’s disease (AD) and ischemic stroke (IS) are common neurological disorders, and the comorbidity of these two brain diseases is often seen. Although AD and IS were regarded as two distinct disease entities, in terms of different etiologies and clinical presentation, recent genome-wide association studies (GWASs) revealed that there were common risk genes between AD and IS, indicating common molecular pathways and their common pathophysiology. In this review, we summarize AD and IS risk single nucleotide polymorphisms (SNPs) and their representative genes from the GWAS Catalog database, and find thirteen common risk genes, but no common risk SNPs. Furthermore, the common molecular pathways associated with these risk gene products are summarized from the GeneCards database and clustered into inflammation and immunity, G protein-coupled receptor, and signal transduction. At least seven of these thirteen genes can be regulated by 23 microRNAs identified from the TargetScan database. Taken together, the imbalance of these molecular pathways may give rise to these two common brain disorders. This review sheds light on the pathogenesis of comorbidity of AD and IS, and provides molecular targets for disease prevention, manipulation, and brain health maintenance.

## 1. Introduction

In the elderly, brain health is mainly jeopardized by dementia or stroke [1]. About 60–80% of dementia cases are Alzheimer’s disease (AD), which is a progressive and irreversible neurodegenerative disorder. Ischemic stroke (IS) accounts for almost 80% of total stroke cases [2], and it is a leading cause of disability and mortality in the elderly worldwide. Pathological studies have shown that the comorbidity of AD and IS is common, infarctions were present in 51.3% of patients with probable AD and 62.5% of patients with possible AD [3]. However, whether these two common brain disorders have the same underlying molecular mechanisms is controversial [4,5,6,7,8].

Clinically, AD is characterized by the inability to recall recent events, changes in personality, and difficulty in solving problems, at the disease’s early stage. AD patients may develop behavioral changes, confusion, disorientation, communication impairment, and diminished social skills at a later stage, and eventually, the patients may have difficulties in speaking, swallowing, and walking. Pathologically, AD is mainly characterized by hippocampal neuronal loss, amyloid β (Aβ) extracellular deposition forming senile plaques, and the formation of intracellular neurofibrillary tangles. Clinically, IS can present with a variety of physical and cognitive manifestations depending on the brain areas affected and associated with neuron lysis and death [9]. Multiple biological processes and molecular pathways are involved in both AD and IS. Vascular dysfunction (hypertension, hyperlipidemia, diabetes, coronary artery disease, etc.) was considered to have significant direct and indirect impacts on neurodegeneration in AD [10], and the presence of hyperlipidemia had a direct influence on the neuronal tau uptake in the entorhinal cortex [11]. The early appearance of vascular dysfunction in the preclinical stage of AD indicates that vascular events might be a part of the cause, rather than a passive contribution, of AD [12]. In addition, AD before IS was considered to be an independent predictor of death in patients under 80 years old [13]. The shared risk factors and protective factors between AD and IS were also identified. The risk factors include low education, sedentary lifestyle, heart diseases, advancing age [14,15], diabetes [16,17], and obesity [18,19], while the protective factors include high levels of psychological well-being, a rich social network, and active engagement in leisure activities [20].

Genetic risk factors play an important susceptible role in both AD and IS [6]. Genome-wide association studies (GWASs) with a large sample size, and their meta-analyses, have identified many AD and IS susceptible single nucleotide polymorphisms (SNPs) and genetic loci [21,22]. Through comparing the causal and susceptible genes of AD and IS by reviewing familial databases and GWAS databases, we aim to identify the common genetic contributors, summarize the common pathways, microRNAs (miRNAs) within the common mapped genes, and eventually provide molecular targets for the improvement of brain health.

## 2. Searching Methodology 

The literature was reviewed by searching the GWAS Catalog database (https://www.ebi.ac.uk/gwas/, accessed on 14 September 2022 from the database’s inception to 14 September 2022. The GWAS Catalog data was extracted from the published GWASs, identified through a weekly PubMed search (https://pubmed.ncbi.nlm.nih.gov/, accessed on 14 September 2022). In the database, the search terms used were: “Alzheimer’s disease” and “ischemic stroke”. The information on risk SNPs and their mapped genes was extracted and aligned. If the SNP was located within a gene, that gene was listed, with multiple overlapping genes separated into several genes. If the gene was intergenic, the upstream and downstream genes were listed.

For familial genetic studies, the vascular neuropathologies of cases carrying familial causative AD genes were reviewed, and genetic variants of AD causative genes in relation to IS were investigated. Vice versa, the AD neuropathologies of cases carrying familial IS genes were reviewed, and genetic variants of IS causative genes in relation to AD were analyzed through a weekly Pubmed search.

**Figure 1:**
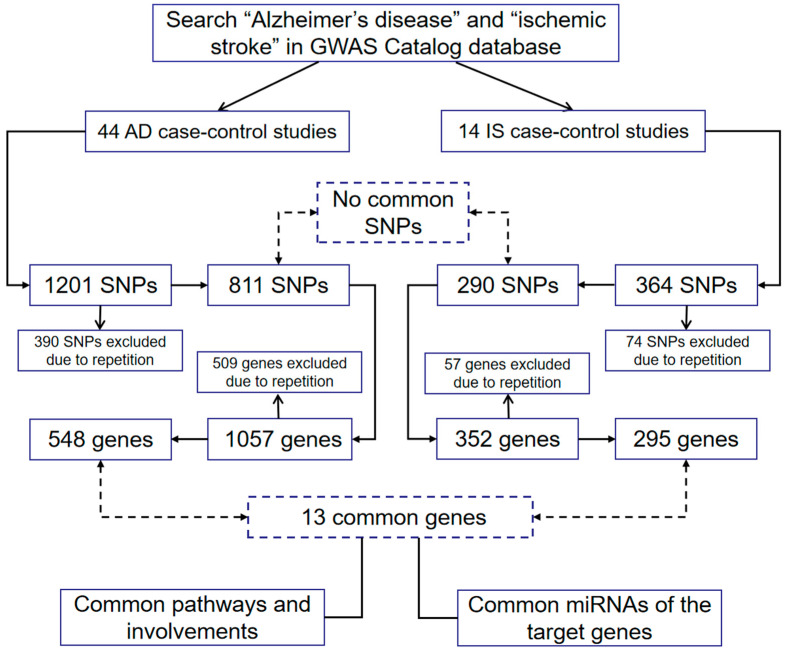
The molecular selection processes. Abbreviation: AD: Alzheimer’s disease; GWAS: genome-wide association study; IS: ischemic stroke; miRNAs: microRNAs; SNP: single nucleotide polymorphism.

Information on the pathways that the gene products were involved in was sourced from the GeneCards database (version 5.12, https://www.genecards.org/, accessed on 17 September 2022). The section “pathways” provides links to pathways according to information extracted from Cell Signaling Technology, R&D Systems, GeneGo (Thomson Reuters), Reactome, Sino Biological, Tocris Bioscience, PharmGKB, Qiagen, GeneTex, BosterBio, MedChemExpress, WikiPathways and PubChem, SuperPathways from PathCards, the protein-protein interaction network was derived based on the STRING database (https://cn.string-db.org/, accessed on 18 September 2022). We identified the miRNAs that regulate the target genes through the TargetScan database (https://www.targetscan.org/vert_80/, accessed on 18 September 2022). The molecular selection processes are shown in a flow chart (Figure 1).

## 3. The Roles of AD or IS Causative Genes in Its Counterpart

*APP*, *PSEN1*, and *PSEN2* genes, are causal genes for AD, which encode proteins called amyloid precursor protein (APP), presenilin1 (PSEN1), and presenilin 2 (PSEN2), respectively. The Apolipoprotein E (*ApoE*) gene is a major player in sporadic AD. The major hereditary IS diseases are cerebral autosomal dominant arteriopathy with subcortical infarcts and leukoencephalopathy (CADASIL), cerebral autosomal recessive arteriopathy/arteriosclorosis with subcortical infarcts and leukoencephalopathy (CARASIL), and Fabry disease, which are caused by mutations in the notch receptor 3 (*Notch3*), htrA serine peptidase 1 (*HTRA1*), and galactosidase alpha (*GLA*) genes, respectively.

### 3.1. AD Causative Genes in IS

No study has found a direct effect of *APP*, *PSEN1*, or *PSEN2* mutations on the risk of IS. However, they may influence the risk of IS through their effects on lipid metabolism [23]. When evaluating the protein oxidation and lipid peroxidation in the brain from knock-in mice expressing mutant human APP and PSEN1, it was observed that knock-in mice displayed increased oxidative stress, which is independent of dietary cholesterol [24]. Lipid peroxidation is closely related to endothelial dysfunction, and finally contributes to atherosclerosis and IS [25]. Statins are widely used drugs that elicit plaque stabilization and reduce inflammation in atherosclerotic plaque, and also reduce Aβ in human neurons through decreasing the generation of soluble APPβ and increasing the levels of full-length APP, indicating that AD and IS may have the same therapeutic target [26].

*ApoE* has been consistently considered as the major genetic risk for sporadic AD and cognitive decline post-IS [27]. The *ApoE* ε4 is associated with an increased number of Aβ plaques, but we did not find *ApoE* related to IS in the GWAS database, although *ApoE* has been shown to be associated with IS risk conditions, such as diabetes [28] and low density lipoprotein cholesterol (LDL-C) [29], which may finally lead to IS.

### 3.2. IS Causative Genes in AD

It has been suggested that Notch3 and HTRA1 are associated with AD [30,31]. Among 5617 participants with AD and 4594 controls, Notch3 rs149307620 allele missense mutation was observed in 10 participants with AD but not in the controls [30]. Notch3 was also an important hit in the gene-based analysis (combined effect of all Notch3 variants) of AD, suggesting its potential role as a modifier of AD [31]. It was found that the T allele of HTRA1 rs2293871 upregulated HTRA1 expression and was associated with an increased risk of AD and AD-by proxy [32]. The deficiency or increase of GLA induced by gene variations has not been shown to be associated with AD.

## 4. Common Susceptibility Genes of AD and IS

We found there were thirteen common mapped genes in AD and IS, although no common risk SNPs were found. The common genes are *ALDH1A2*, *ANKRD22*, *ANTXR1*, *DIO2-AS1*, *HDAC9*, *JH3*, *KCNN3*, *LNC-LBCS*, *MMP3*, *MMP12*, *PCSK6*, *RBMS3*, and *RNU6-909P*. Among them, ten genes are protein coding genes, including *ALDH1A2*, *ANKRD22*, *ANTXR1*, *HDAC9*, *JPH3*, *KCNN3*, *MMP3*, *MMP12*, *PCSK6*, and *RBMS3*. The shared genetic studies of AD and IS are summarized in Table 1. Among the thirteen genes, *HDAC9*, *MMP3*, and *MMP12* have been relatively well studied in AD and IS.

### 4.1. HDAC9

Histone deacetylase 9 (HDAC9) is responsible for the lysine deacetylation residues on the N-terminal part of the core histones. Histone deacetylation is involved in epigenetic repression, and plays a significant role in transcriptional regulation, cell cycle progression, and some developmental events. In AD, the expression of HDAC9 was significantly reduced in the sampled prefrontal and visual cortices [46]. However, the level of blood HDAC9 mRNA expression was increased in AD twins compared to healthy twins [47]. The circRNA HDAC9 (circHDAC9) decreased miRNA (miR)-138 expression, which reversed excessive Aβ production, but circHDAC9 was reduced in AD patients [48]. Thus, in AD, whether the risk allele (A) of rs117756856 is associated with the increased or decreased expression of HDAC9 is not clear. An in vitro study showed that HDAC9 inhibition had neuroprotective effects on IS by inhibiting inflammation [49], which confirmed that depletion of HDAC9 could reduce cerebral injury in experimental stroke. In addition, HDAC9 contributed to brain micro-vessel endothelial cell dysfunction in IS, evidenced by reduced tight-junction proteins’ expression, endothelial cell permeability dysfunction, increased inflammatory responses, and cellular apoptosis [50]. It has also been demonstrated that a higher methylation level of HDAC9 is associated with a lower risk of IS [51]. The risk allele of rs2107595 increased the risk of atherosclerotic stroke through interaction with the *HDAC9* promoter and increased the transcription capacity, which was related to higher *HDAC9* mRNA levels [52]. Pathways associated with the risk alleles of rs2107595 are involved in cholesterol efflux, platelet aggregation, and IL-6 signaling [53]. Thus, the risk alleles of rs2107595, together with rs11984041, rs2023938, and rs71524263, may be associated with an increased expression of HDAC9 contributing to IS.

### 4.2. MMP3 and MMP12

Matrix metalloproteinase 3 (MMP3) and matrix metalloproteinase 12 (MMP12) are two members of the matrix metalloproteinases (MMPs) family, which are both involved in the breakdown of the extracellular matrix (ECM). Microglia surround the Aβ plaques, provoke an inflammatory response, and contribute to neuronal cell loss. Aβ1-42 induces the upregulation of MMP3 and MMP12 in microglia, which further enhances the inflammatory processes and accelerates the progression of AD [54]. The risk alleles of rs12808148 may be associated with the increase of MMP3 and MMP12 in AD. In carotid atherosclerosis plaques, MMP3 and MMP12 may play an important role in the plaque stability, and their low content in plaque is related to a higher risk of ipsilateral stroke [55]. A previous study indicated the causal link between lower serum MMP12 levels and a higher risk of IS [56], and MMP3 exerted a negative effect on the progression of IS [57]. The risk allele of rs72983521 may be associated with MMP3 and MMP12 levels in different sites, and thus is associated with IS.

### 4.3. Other Genes

ANTXR Cell Adhesion Molecule 1 (ANTXR1) is a type I transmembrane protein and a tumor-specific endothelial marker. The higher peptide from ANTXR1 showed a significant ability to discriminate AD patients from healthy controls, which suggested that the risk allele of rs7561207 may increase the risk of AD through enhancing the expression of ANTXR1 [58]. Ankyrin repeat domain 22 (ANKRD22) is a nuclear-encoded mitochondrial membrane protein. The expression of ANKRD22 in AD patients was significantly lower than those of normal controls. However, ANKRD22 stimulated the cytotoxic effect of Aβ and reduced hippocampal neuronal cell viability in an AD cell model [59]. The T allele of rs147285445 may influence the risk of AD differently in vivo and vitro. Proprotein convertase Subtilisin/Kexin type 6 (PCSK6) is a protease in the extracellular matrix and is expressed in many tissues, including the brain. The levels of PCSK6 were increased in the fibrous caps of symptomatic carotid plaques. PCSK6 is possibly involved in the inflammation, ECM remodeling, and dysregulation of smooth muscle cell proliferation in atherosclerosis, and thus could lead to IS [60]. The risk allele of rs528002287 may enhance the expression of PCSK6 and increase the risk of IS. The relationships of the aldehyde dehydrogenase 1 family member A2 (*ALDH1A2*), DIO2 antisense RNA 1 (*DIO2-AS1*), *JH3*, potassium calcium-activated channel subfamily N member 3 (*KCNN3*), long noncoding RNAs bladder and prostate cancer suppressor (*LNC-LBCS*), RNA binding motif single stranded interacting protein 3 (*RBMS3*) and RNA, U6 small nuclear 909, and pseudogene (*RNU6-909P*) genes, and AD, IS were only observed in populational studies and have not been confirmed in molecular studies yet.

**Figure 2:**
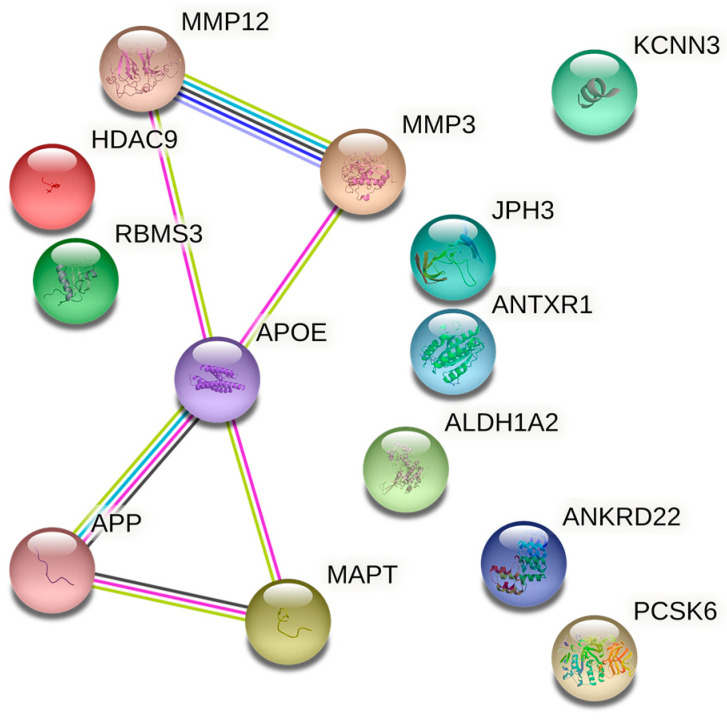
The protein-protein interaction network of APP, MAPT, ApoE, and thirteen common protein coding susceptibility genes products (confidence score ≥ 0.4). MMP3 and MMP12 link with ApoE. Abbreviation: ApoE: apolipoprotein E; APP: amyloid precursor protein; MAPT: microtubule associated protein tau; MMP12: matrix metalloproteinase 12; MMP3: matrix metalloproteinase 3.

To address the common AD and IS risk genes products in AD important molecules, a STRING analysis was performed. The thirteen common AD and IS risk genes in the AD important molecules network were identified. MMP3 and MMP12 links with ApoE, indicating that ApoE may associate with IS through connecting with MMP3 and MMP12 (Figure 2).

## 5. Common Molecular Pathways

We observed three common pathways with at least three gene products related together. The *HDAC9*, *MMP3*, and *MMP12* gene products are involved in the G protein-coupled receptors (GPCR) pathway. Both *HDAC9* and *MMP3* gene products participate in the macrophage-migration inhibitory factor (MIF) mediated glucocorticoid regulation pathway, both *MMP3* and *MMP12* gene products are involved in the transendothelial migration of leukocytes pathway, and the two pathways are both immune related pathways. *MMP3*, *ALDH1A2*, *HDAC9*, and *PCSK6* gene products participate in the signal transduction pathway; *ALDH1A2* and *MMP3* gene products are involved in the estrogen receptor (ESR)-mediated signaling and signaling by nuclear receptors pathways; *ALDH1A2* and *HDAC9* gene products are involved in the ethanol effects on histone modifications pathway; both *MMP3* and *MMP12* gene products participate in the urokinase-type plasminogen activator (uPA) and urokinase plasminogen activator receptor (uPAR)-mediated signaling and UPA-UPAR pathway, and these six pathways are signal transduction pathways (Supplementary Table S1).

### 5.1. Inflammation and Immunity

Immunity is the protection against a disease generated by immunization, previous infection, or other non-immunologic factors. Inflammation plays an important part in immunity. Inflammation is a process of removing damaged cells, infectious microorganisms, and starting to heal. When the trigger of the response is neutralized, immune cells change their activity towards a pro-resolution status via anti-inflammatory signaling. After a proper response, immune cells are recruited to the place where the attack occurs by pro-inflammatory signaling pathways. But when it becomes dysfunctional and chronic, systemic inflammation is an important factor in multiple diseases [61]. Increasing evidence has shown that peripheral and neuroinflammation are the main causes of various neuropsychiatric diseases, including AD and IS.

Neuritis aggravates brain injury, resulting in neuronal degeneration and synaptic dysfunction. Recent studies have shown that inflammation is one of the important factors in the pathogenesis of AD [62]. The type and severity of brain tissue damage is one of the important factors that determines the inflammatory mode of AD. Although the purpose of the initial inflammatory response is to protect the body from the effects of stress factors, if the duration or level of the stimulation is too high, it may cause damage [63]. After Aβ deposition, the pathological adaptations stimulate the release of pro-inflammatory cytokines [interleukin-6 (IL-6), interleukin-1β (IL-1β) or tumor necrosis factor-α (TNF-α)] and other pro-inflammatory molecules [macrophage inflammatory protein, monocyte chemoattractant protein, coagulation factor, reactive oxygen species (ROS), nitric oxide, protease, protease inhibitor, etc.], and some prostaglandin, thromboxane, leukotriene, and C-reactive protein (CRP) from glial cells [64]. Activated microglia respond to the Aβ, resulting in migration to the plaques as well as phagocytosis of the Aβ. However, when the microglia become enlarged, or after prolonged periods, they are no longer able to phagocytose the Aβ. Then peripheral macrophages may migrate to Aβ plaque deposition to clear the Aβ. However, peripheral macrophages recruitment into the brain is likely to exacerbate the effects of sustained inflammation and results in an exacerbation of AD pathology [65]. The deterioration of the environment will lead to additional Aβ accumulation and pro-inflammatory molecules [64], which will release reactive substances such as nitric oxide, proteolytic enzymes, excitatory amino acids and complementary factors, and cause damage to the adjacent neurons [66,67]. Although inflammation is mainly considered to be what happens after IS, it also has a close relationship with atherosclerosis, which is a key risk factor for IS. Numerous studies have shown that atherosclerosis is initiated by endothelial injury or LDLs accumulation within the arterial vascular wall, which generally involves in oxidization or modification. These modified or oxidized LDLs, and low-grade inflammation are caused by small endothelial injuries, activate innate and adaptive immune responses. These immune responses play important roles in the development of atherosclerosis [68]. Monocytes/macrophages, neutrophils, B lymphocytes, and T lymphocytes are the major cell subtypes in the components of atherosclerosis [69]. The increased levels of IL-6, CRP and lipoprotein-associated phospholipase A2 (Lp-PLA2) are related to an increased risk of IS [70].

MIF is a cytokine released from T-lymphocytes and macrophages stimulated by glucocorticoids. MIF counter-regulates the inhibitory effects of glucocorticoids on pro-inflammatory cytokines (IL-6, IL-8, IL-1β and TNF-α), and overcomes glucocorticoid’s inhibition of T-cell proliferation [71]. MIF may exacerbate the effects of sustained inflammation and leads to additional Aβ accumulation. In addition, T-lymphocytes are major components of atherosclerosis [72]. Increased levels of IL-6 are related to an increased risk of IS. Thus, MIF mediated glucocorticoid regulation is connected with an increased risk of both AD and IS. In the functional enrichment analysis of the 49 differently expressed genes among AD and control brain samples, transendothelial migration of leukocytes was enriched in the AD group [73]. Transendothelial leukocyte migration is a key step in the progression of vascular inflammation, the underlying molecular pathogenesis of atherosclerosis [74]. Moreover, the differently expressed long noncoding RNAs (lncRNAs) in IS are also mainly related to transendothelial leukocyte migration [75]. These indicate that transendothelial leukocyte migration is closely associated with both AD and IS.

**Figure 3:**
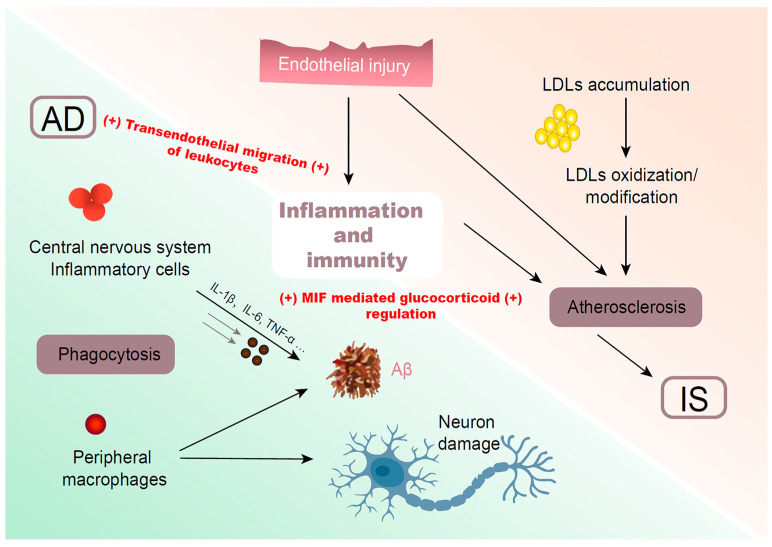
The involvements of inflammation and immunity in AD and IS. Abbreviation: Aβ: amyloid β; AD: Alzheimer’s disease; IL-1β: interleukin-1β; IL-6: interleukin-6; IS: ischemic stroke; LDL: low density lipoprotein; MIF: macrophage-migration inhibitory factor; TNF-α: tumor necrosis factor-α; +: promote the progression.

The inflammatory responses are initiated locally under aberrant local conditions in both AD and IS, and elicited by the immune system (Figure 3). Therefore, AD and IS may have common biological pathways involved in the immune system.

### 5.2. GPCR

**Figure 4:**
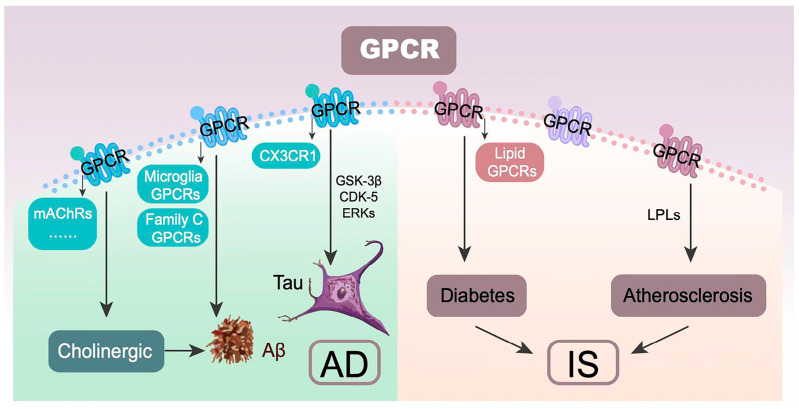
The involvements of GPCRs in AD and IS. Abbreviation: Aβ: amyloid β; AD: Alzheimer’s disease; CDK-5: cyclin-dependent kinase-5; CX3CR1: CX3C chemokine receptor 1; ERKs: extracellular signal-regulated kinases; GPCR: G protein-coupled receptor; GSK-3β: glycogen synthase kinase-3β; IS: ischemic stroke; LPL: lysophospholipids; mAchRs: muscarinic acetylcholine receptors.

GPCRs are the largest membrane protein family of seven-transmembrane receptors in humans. They are involved in neuronal signal transduction in response to various extracellular signals such as hormones and neurotransmitters [76]. GPCRs may participate in AD pathology through three aspects: the amyloid hypothesis, the tau hypothesis, and the cholinergic hypothesis [77]. Microglia express several GPCRs to regulate microglial activation and its polarization status. Microglial GPCRs are involved in Aβ generation, degradation, clearance, and trigger multiple inflammatory pathways in response to Aβ [78]. Family C GPCRs also play important roles in Aβ. Family C of GPCRs contains the calcium-sensing receptor (CaSR), GABAB receptors (GABABRs), and other receptors. CaSRs are involved in the neurotransmitter system of human cortical astrocytes and neurons in vitro. The specific binding of Aβs to CaSRs hinders the release of soluble APP-α peptide and kills human cortical neurons [23]. GABABRs block the Aβ peptides’ synthesis and prevent neuronal hyperexcitability [79]. In AD, GPCRs are involved in tau phosphorylation via various downstream kinases including glycogen synthase kinase-3β (GSK-3β), cyclin-dependent kinase-5 (CDK-5), and extracellular signal-regulated kinases (ERKs) signaling cascade [80,81,82]. CX3C chemokine receptor 1 (CX3CR1) receptor is a microglia chemokine GPCR. Its binding and interaction with tau lead to the internalization of tau into microglia [83]. These GPCRs both promote and inhibit tau phosphorylation. In the cholinergic hypothesis, cholinergic dysfunction is characterized by reduced acetylcholine release and impaired coupling of muscarinic acetylcholine receptors (mAChRs) to heterotrimeric guanosine triphosphate (GTP)-binding proteins (G proteins). Cholinergic dysfunction is also associated with Aβ accumulation [84]. Atherosclerosis and type 2 diabetes are the risk factors for IS. GPCRs influence the risk of IS by affecting its risk factors. Lysophospholipids (LPLs) are second-generation bioactive lipid-derived signaling molecules. GPCRs mediate the biological effects of LPLs in the development of atherosclerosis [85]. β-cells and enteroendocrine cells are essential cells for insulin secretion modulation through expressing numerous GPCRs. GPCRs specific for free fatty acid ligands (lipid GPCRs) are the target for the treatment of type 2 diabetes because of their function in islet and gut hormone secretion [86]. Therefore, different GPCRs may have different influences on AD and IS (Figure 4).

### 5.3. Signal Transduction

The occurrence of both AD and IS are tightly regulated by a multitude of signal transduction pathways. Nuclear receptors generally act as ligand-activated transcription factors [87]. They regulate gene expression through binding to the specific ligand, and present as therapeutic targets in AD and IS. The receptor interacting protein-140 (RIP140) is known as a cofactor for some nuclear receptors. The overexpression of RIP140 was shown to reduce the generation of Aβ by decreasing the transcription of β-APP cleaving enzyme (BACE1) [88]. Elevated RIP140 also increases the risk of insulin resistance and atherosclerosis, which are risk factors of IS [89]. Retinoids binds to the specific nuclear receptor, such as retinoid X receptors (RXRs) and retinoic acid receptors, to regulate the expression of a variety of genes that code for enzymes, receptors, neuropeptide hormone, etc., which are responsible for slowing down the accumulation of amyloids, reducing neurodegeneration, and preventing pathogenesis of AD [90]. RXRs also negatively regulate the platelet functional responses and thrombus formation, which might delay the onset of IS [91]. Liver X receptor (LXR), also known as nuclear receptor subfamily 1 group H member 3 (NR1H3), is a member of the nuclear receptor superfamily of ligand-activated transcription factors, and plays a central role in the transcriptional control of cholesterol homeostasis. The C allele of rs7120118 of *NR1H3* gene was shown to reduce the risk of AD, and the soluble Aβ42 levels were significantly reduced in the temporal cortex of patients with the CC genotype [92]. In an AD mice model, LXR activation restored microvascular morphology through decreasing tortuosity and increasing length, which were associated with decreased deposition of perivascular Aβ [93]. The TT genotype of LXRα rs2279238 is significantly associated with advanced carotid atherosclerosis, suggesting that this polymorphism may act as a genetic risk factor for atherosclerotic stroke [94]. Ex vivo LXR agonist treatment decreases early atherosclerosis in LDL receptor-deficient mice through inhibiting monocyte to endothelial adhesion [95]. In addition, the deficiency of LXR led to an increase in atherosclerosis, with enhanced inflammation in foam cells of atherosclerotic plaques [96]. Peroxisome proliferator-activated receptor γ (PPAR-γ) is a nuclear receptor that plays a crucial role in glucose and lipid homeostasis in the central nervous system. The PPAR-γ agonist pioglitazone significantly increased PPAR-γ expression, lowered amyloid-β levels, and improved the antioxidative capacity in the cortex of AD mice [97]. The PPAR-γ gene rs1801282 GG genotype may be associated with an increased risk of IS [98]. The PPAR-γ agonist improved the dyslipidemic profile and inflammatory status in atherosclerotic lesions in rats [99]. The nuclear receptor subfamily 4A 2 (NR4A2, also known as Nurr1) plays important roles in diverse brain functions and its overexpression alleviated AD pathology changes, including Aβ deposition and neuronal loss [100] in AD mice. The expression of nuclear receptor subfamily 4 group A member 1 (NR4A1, also known as Nur77) was significantly increased in the hippocampus of AD mice; the overexpression of NR4A1 promoted amyloidogenesis and accelerated tau hyperphosphorylation [101]. The absence of Nur77 in macrophages led to upregulated toll-like receptor signaling, and imbalanced macrophage polarization toward the proinflammatory M1 phenotype, indicating that Nur77 is an important target for modulating the inflammatory phenotype of macrophages and regulation of atherogenesis [102].

In AD hypothalamic medial mamillary (MMN), a higher nuclear ESRα intensity was significantly associated with larger nuclear and perikaryal sizes, indicating nuclear ESRα may mediate extra activation in MMN that acts as a unique brain area to prevent neurodegeneration [103]. In AD human brain tissue, ESRα co-localized with neurofibrillary pathology, and their interaction interrupted estrogen signaling, demonstrating that sequestration of ESRα by tau pathology decreased the neuroprotective role of estrogen in AD [104]. In both female aorta surgical sample and bilateral ovariectomized female *ApoE* -/- mice samples, assay results indicated that estrogen prevented atherosclerosis through upregulating ESRα expression to induce ESRα-mediated activation of autophagy and reduce inflammation and cell pyroptosis [105]. In addition, in an ovariectomized *ApoE* -/- mice model, it was demonstrated that ESRα inhibited the synthesis and secretion of proprotein convertase subtilisin/kexin type 9 (PCSK9) and subsequently lowered the accumulation of cholesterol and triglyceride to prevent post-menopausal atherosclerosis [106]. Hyperinsulinemia and insulin resistance are important causes of atherosclerosis. Insulin indirectly reduced the expression of ESRα, and thus interfered with estrogen regulation of vascular smooth muscle cells’ proliferation, leading to atherosclerosis [107].

Urokinase-type plasminogen gene rs2227564 C-positive genotype (CC+CT) has been reported to associate with a higher risk of developing AD [108]. Neuronal uPA protected the synapse from the deleterious effects of soluble Aβ. However, in the frontal cortex of an AD human brain and 5xFAD mice, uPA was decreased, leaving the deleterious effects of Aβ on the synapse unaffected [109]. UPAR was highly expressed in human symptomatic carotid endarterectomies, and mainly found in ruptured plaque segments, suggesting that UPAR may be connected with plaque rupture in the progression of symptomatic atherosclerotic lesions [110]. UPA stimulated the differentiation of monocytes into macrophages, prolonged the macrophage survival in the atherosclerotic lesion, increased lesion cellularity, and eventually accelerated lesion development [111]. The role of ethanol effects on histone modifications in AD and IS has not been reported.

## 6. Common miRNAs

At least nine of these thirteen genes can be regulated by 23 miRNAs identified from the TargetScan database. Among the 23 miRNAs, four miRNAs regulate eight genes, including hsa-miR-204-5p, hsa-miR-211-5p, hsa-miR-548c-3p, and hsa-miR-660-3p; and the other miRNAs regulate seven genes. Among them, we found that thirteen miRNAs were reported through a Pubmed search using the key words of each miRNA (Supplementary Table S2). However, only two miRNAs were reported to be related to AD and/or IS. Hsa-miR-204-5p is the product of the *MIR204* gene, regulating *ALDH1A2*, *ANKRD22*, *ANTXR1*, *HDAC9*, *JH3*, *KCNN3*, *MMP3*, and *RBMS3*. Hsa-miR-664b-3p is the product of *MIR664B*, regulating *ALDH1A2*, *ANKRD22*, *HDAC9*, *KCNN3*, *MMP3*, *MMP12*, *PCSK6*, and *RBMS3*. Heavy metals like Pb acetate increased the expression of has-miR-204-5p, deteriorated the cognitive functions, and were associated with the overexpression of tau via pathways of neurodegeneration-multiple diseases [112]. Has-miR-204-5p also participated in mixed B vitamins’ better cognitive functions in AD, through linking to both mixed B vitamins and cognitive function related genes [113]. Hsa-miR-664b-3p was negatively associated with lead exposure and was decreased in AD human brain tissue compared with controls, and its target genes participated in potentially AD relevant pathways [114]. It was upregulated in human vascular smooth muscle cells (hVSMCs) during replicative senescence. Although hsa-miR-664b-3p was not functionally well annotated in hVSMCs to date, it may play a role in the adaptive immune system and toll-like receptor signaling 7/8/9 [115]. It may be associated with vascular aging and atherosclerosis, contributing to IS. Hsa-miR-664b-3p also regulates *APP*, *PSEN1*, *MAPT*, and *HTRA1* (Supplementary Table S3).

## 7. Advantages and Limitations

Compared with other studies (Table 2), our study has summarized all up-to-date AD and IS GWAS studies to identify the common risk SNPs and related genes. In addition, we clustered the common molecular pathways indicated by the shared genes into three common potential pathogeneses for AD and IS. Finally, we searched for the miRNAs that regulated the commonly susceptible genes and identified 23 miRNAs that might regulate both AD and IS. There are some limitations in our study. First, our study was only based on GWAS datasets, so there might be other common susceptibility genes not covered in this review. Second, other types of stroke, such as intracerebral hemorrhage, small vessel disease, etc., might have their own specific genetic factors that are associated with AD, but they were not covered in this review.

In this study, we used genetic data from the GWAS Catalog database and PubMed to determine whether the same genetic loci contribute to AD and IS. We identified thirteen common risk genes, which are involved in three common molecular pathways: immunity, GPCR, and signaling transduction pathways. Furthermore, we identified 23 miRNAs that regulated at least seven of the common risk genes. In summary, there are some common genetic factors and pathways shared by AD and IS, which might be the molecular targets for maintaining good brain health jeopardized by AD or IS.

## Figures and Tables

**Table 1:** Common susceptibility genes and their related SNPs in AD and IS.

Mapped Gene	Region	SNP	Position Relative to Gene	Risk Allele	*p* Value	Risk to	Reference
*ALDH1A2*	15q21.3	rs4775044	intron variant	N.A	4.0 × 10^−6^	AD	Schwartzentruber et al., 2021 [33]
		rs4471613	intron variant	A	5.0 × 10^−7^	IS	Carty et al., 2015 [34]
*ANKRD22*	10q23.31	rs147285445	intron variant	T	4.0 × 10^−6^	AD	Mez et al., 2017 [35]
		rs11202867	intron variant	N.A	1.0 × 10^−5^	IS	Kumar et al., 2021 [36]
*ANTXR1*	2p13.3	rs7561207	intron variant	N.A	4.0 × 10^−6^	AD	Nazarian et al., 2019 [37]
		rs149587156	intergenic variant	N.A	5.0 × 10^−6^	IS	Kumar et al., 2021 [36]
*DIO2-AS1*	14q31.1	rs7155666	intron variant	A	8.0 × 10^−10^	AD	Chung et al., 2022 [38]
		rs11846182	intron variant	T	9.0 × 10^−6^	IS	Lee et al., 2017 [39]
*HDAC9*	7p21.1	rs117756856	intron variant	A	9.0 × 10^−6^	AD	Mez et al., 2017 [35]
		rs11984041	intron variant	T	5.0 × 10^−9^	IS	Network NSG. 2016 [40]
		rs2023938	3’prime UTR variant	G	8.0 × 10^−7^	IS	Malik et al., 2017 [41]
		rs71524263	intron variant	N.A	2.0 × 10^−12^	IS	Traylor et al., 2017 [42]
		rs2107595	regulatory region variant	N.A	4.0 × 10^−15^	IS	Malik et al., 2018 [22]
*JPH3*	16q24.2	rs117760708	non coding transcript exon variant	T	3.0 × 10^−6^	AD	Mez et al., 2017 [35]
		rs12445022	intergenic variant	A	9.0 × 10^−8^	IS	Traylor et al., 2021 [43]
*KCNN3*	1q21.3	rs16830122	intron variant	A	2.0 × 10^−6^	AD	Jun et al., 2016 [44]
		rs114812453	intergenic variant	N.A	3.0 × 10^−7^	IS	Kumar et al., 2021 [36]
*LNC-LBCS*	6p22.3	rs62402815	intron variant	N.A	2.0 × 10^−6^	AD	Nazarian et al., 2019 [37]
		rs9348394	intron variant	N.A	5.0 × 10^−6^	IS	Kumar et al., 2021 [36]
*MMP12*	11q22.2	rs12808148	intergenic variant	N.A	1.0 × 10^−6^	AD	Kamboh et al., 2012 [45]
		rs72983521	intergenic variant	N.A	3.0 × 10^−8^	IS	Malik et al., 2018 [22]
*MMP3*	11q22.2	rs12808148	intergenic variant	N.A	1.0 × 10^−6^	AD	Kamboh et al., 2012 [45]
		rs72983521	intergenic variant	N.A	5.0 × 10^−8^	IS	Malik et al., 2018 [22]
*PCSK6*	15q26.3	rs146322114	intron variant	A	2.0 × 10^−6^	AD	Mez et al., 2017 [35]
		rs528002287	intron variant	N.A	6.0 × 10^−6^	IS	Kumar et al., 2021 [36]
*RBMS3*	3p24.1	rs17022021	intron variant	T	8.0 × 10^−6^	AD	Mez et al., 2017 [35]
		rs115182009	intron variant	N.A	9.0 × 10^−7^	IS	Kumar et al., 2021 [36]
*RNU6-909P*	5p14.1	rs150631144	intron variant	T	1.0 × 10^−7^	AD	Mez et al., 2017 [35]
		rs13354619	intron variant	N.A	2.0 × 10^−6^	IS	Kumar et al., 2021 [36]

Abbreviation: AD: Alzheimer’s disease; IS: ischemic stroke; N.A: not available; SNP: single nucleotide polymorphisms.

**Table 2:** The studies about the common genetic factors, pathways and molecular mechanisms between AD and IS.

No.	Reference	AD and IS		Common Molecular Pathways Involved	Others
		NoSG	NoSS		
1	Traylor et al. 2016 [4]	0	0	Phospholipid efflux, cholesterol efflux, reverse cholesterol transport, negative regulation of nuclear factor kappa B (NF-κB) transcription factor activity (AD and small vessel disease)	One region (ATP5H/KCTD2/ICT1) associated with both AD and small vessel disease
2	Cui et al. 2018 [5]	/	/	Glioma, toll-like receptor signaling pathway, non-small cell lung cancer, natural killer cell mediated cytotoxicity, phospholipase D signaling pathway, hepatitis B, cadherin signaling pathway, wnt signaling pathway, immunoregulatory interactions between a lymphoid and a non-lymphoid cell, synthesis of PIPs at the plasma membrane, cooperation of PDCL (PhLP1) and TRiC/CCT in G-protein beta folding, PI metabolism, signaling pathways in glioblastoma	56 biological processes, 95 cellular components, and 28 molecular functions
3	Wei et al. 2019 [6]	16	/	Immune system	/
4	Rahman MR et al. 2019 [7]	22	/	Alcoholism, MAPK signaling, glycine metabolism, serine metabolism, threonine metabolism	Transcriptional regulator: SPIB, SMAD3, and SOX2
5	Wang T, et al.2020 [8]	0	0	Different types of stroke, including any stroke, any ischemic stroke, large artery stroke, and cardio-embolic stroke would not be causally associated with AD risk	/
6	This study	13	0	MIF mediated glucocorticoid regulation, transendothelial migration of leukocytes, GPCR pathway, signal transduction, signaling by nuclear receptors, ESR-mediated signaling, ethanol effects on histone modifications, urokinase-type plasminogen activator (uPA) and uPAR-mediated signaling, UPA-UPAR pathway	23 miRNAs regulate more than seven common risk genes

Abbreviation: ATP5H: adenosine triphosphate (ATP) synthase, H+ transporting, mitochondrial F0; ESR: estrogen receptor; GPCR: G protein-coupled receptors; ICT1: Immature colon carcinoma transcript 1; KCTD2: Potassium channel tetramerization domain-containing protein 2; MAPK: Mitogen-activated protein kinase; MIF: macrophage-migration inhibitory factor; miRNA: microRNA; NoSG: Number of Shared Genes, NoSS: Number of Shared SNP, PDCL: Phosducin like; PhLP1: PH domain and leucine rich repeat protein phosphatase 1; PI : Phosphatidylinositol; SMAD3: SMAD family member 3; SOX2: SRY-Box transcription factor 2; SPIB: Spi-B Transcription factor; TRiC/CCT: TCP1 ring complex/chaperonin containing TCP1 complex; uPAR: urokinase plasminogen activator receptor; /: not reported.8. Summary.

## Data Availability

Data is contained within the article.

## Supplementary Materials

The following supporting information can be downloaded at: https://www.mdpi.com/article/10.3390/genes14020353/s1, Table S1: Common molecular pathways related to the common susceptibility genes; Table S2: Summary of diseases/traits related to the 23 miRNAs in the literature [112,113,114,115,116,117,118,119,120,121,122,123,124,125,126,127,128,129,130,131,132,133,134,135,136,137,138,139,140,141,142,143,144,145,146,147,148,149,150,151,152,153,154,155,156,157,158,159,160,161,162,163,164,165,166,167,168,169,170,171,172,173,174,175,176,177,178,179,180]; Table S3: The common miRNAs that regulate the shared susceptibility genes of AD and IS.
