# Supplementary material for: Common Genetic Factors and Pathways in Alzheimer’s Disease and Ischemic Stroke: Evidences from GWAS
Source: Genes (Basel). 2023 Jan 30;14(2):353. doi: 10.3390/genes14020353 (PMC9957001; doi:10.3390/genes14020353)
Supplement: Supplementary file 1 [file genes-14-00353-s001.zip › genes-2126527-supplementary.pdf]

Supplementary Table S1. Common molecular pathways related to the common susceptibility genes.

<table>
<tr><th></th><th>Pathways</th><th colspan="2">Genetic products involved</th></tr>
<tr><td rowspan="2">Inflammation and immunity</td><td>MIF mediated glucocorticoid regulation</td><td>HDAC9</td><td>MMP3</td></tr>
<tr><td>transendothelial migration of leukocytes</td><td>MMP12</td><td>MMP3</td></tr>
<tr><td rowspan="2">GPCR</td><td rowspan="2">GPCR pathway</td><td>HDAC9</td><td>MMP12</td></tr>
<tr><td>MMP3</td><td>/</td></tr>
<tr><td rowspan="7">Signal transduction</td><td rowspan="2">Signal transduction</td><td>ALDH1A2</td><td>HDAC9</td></tr>
<tr><td>MMP3</td><td>PCSK6</td></tr>
<tr><td>Signaling by nuclear receptors</td><td>ALDH1A2</td><td>MMP3</td></tr>
<tr><td>ESR-mediated signaling</td><td>ALDH1A2</td><td>MMP3</td></tr>
<tr><td>Ethanol effects on histone modifications</td><td>ALDH1A2</td><td>HDAC9</td></tr>
<tr><td>Urokinase-type plasminogen activator (uPA) and uPAR-mediated signaling</td><td>MMP12</td><td>MMP3</td></tr>
<tr><td>UPA-UPAR pathway</td><td>MMP12</td><td>MMP3</td></tr>
</table>

Abbreviation: ESR: estrogen receptor; GPCR: G protein-coupled receptor; MIF: macrophage-migration inhibitory factor; uPAR: urokinase plasminogen activator receptor; /: blank cell

Supplementary Table S2. Summary of diseases/traits related to the 23 miRNAs in the literature.

**MiRNA**	**Reported diseases/traits**	**Refs**
**hsa-miR-204-5p**	Adenoid cystic carcinoma of the salivary gland, Alzheimer's disease, axon regeneration, breast cancer, cell-free bone regeneration, diabetic cataract lenses, diabetic retinopathy, depression, gastric cancer, hepatitis C virus infection, human cancers, in vitro fertilization-embryo transfer, laryngeal squamous cell carcinoma, metastatic renal cell carcinoma, periodontitis and periimplantitis, recurrent spontaneous abortion, rheumatic carditis, thyroid carcinoma	[1-20]
**hsa-miR-211-5p**	Cervical cancer cells, open-angle glaucoma, preeclampsia	[21-23]
**hsa-miR-548c-3p**	Alcoholic cardiomyopathy, hypopharyngeal cancer, metabolic syndrome, metastatic prostate cancers	[24-27]
**hsa-miR-660-3p**	Liver fibrosis, osteosarcoma	[28-29]
hsa-miR-10b-3p	Colorectal cancer, hepatocellular carcinoma	[30-31]

hsa-miR-1250-3p	/	
hsa-miR-20a-3p	Glioblastoma, knee osteoarthritis, laryngeal squamous cell carcinoma	[32-34]
hsa-miR-3182	Chronic kidney disease, cleft palate, colorectal cancer, kidney disease	[35-40]
hsa-miR-3662	Adult T-cell leukemia/lymphoma, breast cancer, carcinomas, hematopoiesis and acute myeloid leukemia, hepatocellular carcinoma, lung cancer, lung adenocarcinoma, pancreatic cancer, recurrent miscarriage, thyroid eye disease	[41-55]
hsa-miR-3688-3p	/	
hsa-miR-3919	/	
hsa-miR-4524b-3p	/	
hsa-miR-4755-5p	/	
hsa-miR-4775	Alport syndrome, intervertebral disc degeneration	[56-57]
hsa-miR-5006-3p	/	
hsa-miR-5692a	Livers of type 2 diabetes mellitus, malignancies	[58-59]
hsa-miR-579-3p	Coronavirus replication, prostate cancer, fear and anxiety, metabolic syndrome, Parkinson's disease, sperm	[25,60–64]
hsa-miR-664b-3p	Alzheimer's disease, breast cancer, coronavirus replication, ileocecal adenocarcinoma, vascular disease	[60,65–68]
hsa-miR-6749-3p	/	
hsa-miR-7152-5p	/	
hsa-miR-7515	/	
hsa-miR-8063	Cell migration	[69]
hsa-miR-95-5p	/	

Abbreviation: miRNA: microRNA; /: not available

Supplementary Table S3. The common miRNAs that regulate the shared susceptibility genes of AD and IS.

	The common susceptibility genes of AD and IS									Dementia causative genes			IS causative genes
	ALDH1A2	*ANKRD22*	*ANTXR1*	*HDAC9*	*JPH3*	*KCNN3*	*MMP3*	*PCSK6*	*RBMS3*	*PSEN1/2*	*APP*	*MAPT*	*Notch3/HTRA1/GLA*
**hsa-miR-204-5p**	*	*	*	*	*	*	*		*			*	*
**hsa-miR-211-5p**	*	*	*	*	*	*	*		*				*
**hsa-miR-548c-3p**		*	*	*	*	*	*	*	*	*	*		*
**hsa-miR-660-3p**	*	*	*	*	*	*		*	*	*		*	*
hsa-miR-10b-3p	*	*	*	*		*	*		*				
hsa-miR-1250-3p	*	*	*	*		*		*	*		*		
hsa-miR-20a-3p	*	*	*	*		*		*	*				*
hsa-miR-3182		*	*	*	*	*		*	*				*
hsa-miR-3662	*	*	*	*			*	*	*		*		
hsa-miR-3688-3p	*	*	*	*	*	*			*				*
hsa-miR-3919	*	*	*		*	*		*	*				*
hsa-miR-4524b-3p	*	*	*	*	*	*			*			*	
hsa-miR-4755-5p		*	*	*	*	*	*		*		*		
hsa-miR-4775		*	*	*	*	*		*	*		*	*	*
hsa-miR-5006-3p		*	*	*	*	*	*		*		*	*	
hsa-miR-5692a	*	*	*			*	*	*	*		*		*
hsa-miR-579-3p	*	*		*		*	*	*	*		*		
hsa-miR-664b-3p	*	*		*		*	*	*	*	*	*		*
hsa-miR-6749-3p	*	*	*	*	*	*			*	*			
hsa-miR-7152-5p		*	*	*	*	*		*	*	*			*
hsa-miR-7515	*	*	*	*		*		*	*		*		*
hsa-miR-8063	*	*	*	*		*		*	*	*		*	
hsa-miR-95-5p		*	*	*	*	*		*	*	*		*	

Abbreviation: AD: Alzheimer's disease; IS: ischemic stroke; miRNA: microRNA

References

1. Nguyen, H.D. Resveratrol, Endocrine Disrupting Chemicals, Neurodegenerative Diseases and Depression: Genes, Transcription Factors, microRNAs, and Sponges Involved. *Neurochem Res* **2022**, doi:10.1007/s11064-022-03787-7.
2. Shi, J.; Li, L. circKMT2E Protect Retina from Early Diabetic Retinopathy through SIRT1 Signaling Pathway via Sponging miR-204-5p. *Comput Math Methods Med* **2022**, *2022*, 7188193, doi:10.1155/2022/7188193.
3. Nguyen, H.D.; Kim, M.S. The role of mixed B vitamin intakes on cognitive performance: Modeling, genes and miRNAs involved. *J Psychiatr Res* **2022**, *152*, 38-56, doi:10.1016/j.jpsychires.2022.06.006.
4. Liu, X.; Wu, J.; Nie, H.; Zhu, X.; Song, G.; Han, L.; Qin, W. Comprehensive Analysis of circRNAs, miRNAs, and mRNAs Expression Profiles and ceRNA Networks in Decidua of Unexplained Recurrent Spontaneous Abortion. *Front Genet* **2022**, *13*, 858641, doi:10.3389/fgene.2022.858641.
5. Nguyen, H.D.; Kim, M.S. Exposure to a mixture of heavy metals induces cognitive impairment: Genes and microRNAs involved. *Toxicology* **2022**, *471*, 153164, doi:10.1016/j.tox.2022.153164.
6. Tang, Y.F.; Wu, W.J.; Zhang, J.Y.; Zhang, J. Reconstruction and analysis of the aberrant lncRNA-miRNA-mRNA network based on competitive endogenous RNA in adenoid cystic carcinoma of the salivary gland. *Transl Cancer Res* **2021**, *10*, 5133-5149, doi:10.21037/tcr-21-1771.
7. Yang, S.; Zheng, W.; Yang, C.; Zu, R.; Ran, S.; Wu, H.; Mu, M.; Sun, S.; Zhang, N.; Thorne, R.F.; et al. Integrated Analysis of Hub Genes and MicroRNAs in Human Placental Tissues from In Vitro Fertilization-Embryo Transfer. *Front Endocrinol (Lausanne)* **2021**, *12*, 774997, doi:10.3389/fendo.2021.774997.
8. Zhang, F.; Yu, X.; Lin, Z.; Wang, X.; Gao, T.; Teng, D.; Teng, W. Using Tumor-Infiltrating Immune Cells and a ceRNA Network Model to Construct a Prognostic Analysis Model of Thyroid Carcinoma. *Front Oncol* **2021**, *11*, 658165, doi:10.3389/fonc.2021.658165.
9. Liu, X.; Wang, P.; Teng, X.; Zhang, Z.; Song, S. Comprehensive Analysis of Expression Regulation for RNA m6A Regulators With Clinical Significance in Human Cancers. *Front Oncol* **2021**, *11*, 624395, doi:10.3389/fonc.2021.624395.
10. Hao, M.; Liu, W.; Ding, C.; Peng, X.; Zhang, Y.; Chen, H.; Dong, L.; Liu, X.; Zhao, Y.; Chen, X.; et al. Identification of hub genes and small molecule therapeutic drugs related to breast cancer with comprehensive bioinformatics analysis. *PeerJ* **2020**, *8*, e9946, doi:10.7717/peerj.9946.
11. Zhai, M.; Zhu, Y.; Yang, M.; Mao, C. Human Mesenchymal Stem Cell Derived Exosomes Enhance Cell-Free Bone Regeneration by Altering Their miRNAs Profiles. *Adv Sci (Weinh)* **2020**, *7*, 2001334, doi:10.1002/advs.202001334.
12. Zhou, H.; Chen, D.; Xie, G.; Li, J.; Tang, J.; Tang, L. LncRNA-mediated ceRNA network was identified as a crucial determinant of differential effects in periodontitis and periimplantitis by high-throughput sequencing. *Clin Implant Dent Relat Res* **2020**, *22*, 424-450, doi:10.1111/cid.12911.
13. Ma, T.; Liu, A.; Xu, D.; Zhang, T. Mechanisms underlying the promotion of osteosarcoma cell proliferation and invasion by lncRNA PBB12. *Oncol Rep* **2020**, *43*, 736-746, doi:10.3892/or.2019.7451.
14. Kondo, Y.; Kogure, T.; Ninomiya, M.; Fukuda, R.; Monma, N.; Ikeo, K.; Tanaka, Y. The reduction of miR146b-5p in monocytes and T cells could contribute to the immunopathogenesis of hepatitis C virus infection. *Sci Rep* **2019**, *9*, 13393, doi:10.1038/s41598-019-49706-9.
15. Fan, C.; Liu, X.; Li, W.; Wang, H.; Teng, Y.; Ren, J.; Huang, Y. Circular RNA circ KMT2E is up-regulated in diabetic cataract lenses and is associated with miR-204-5p sponge function. *Gene* **2019**, *710*, 170-177, doi:10.1016/j.gene.2019.05.054.
16. Gumus, G.; Giray, D.; Bobusoglu, O.; Tamer, L.; Karpuz, D.; Hallioglu, O. MicroRNA values in children with rheumatic carditis: a preliminary study. *Rheumatol Int* **2018**, *38*, 1199-1205, doi:10.1007/s00296-018-4069-2.
17. Su, L.N.; Song, X.Q.; Xue, Z.X.; Zheng, C.Q.; Yin, H.F.; Wei, H.P. Network analysis of microRNAs, transcription factors, and target genes involved in axon regeneration. *J Zhejiang Univ Sci B* **2018**, *19*, 293-304, doi:10.1631/jzus.B1700179.
18. Gao, W.; Wu, Y.; He, X.; Zhang, C.; Zhu, M.; Chen, B.; Liu, Q.; Qu, X.; Li, W.; Wen, S.; et al. MicroRNA-204-5p inhibits invasion and metastasis of laryngeal squamous cell carcinoma by suppressing forkhead box C1. *J Cancer* **2017**, *8*, 2356-2368, doi:10.7150/jca.19470.
19. Zhang, T.; Liu, C.; Huang, S.; Ma, Y.; Fang, J.; Chen, Y. A Downmodulated MicroRNA Profiling in Patients with Gastric Cancer. *Gastroenterol Res Pract* **2017**, *2017*, 1526981, doi:10.1155/2017/1526981.
20. Zhu, J.; Ma, X.; Zhang, Y.; Ni, D.; Ai, Q.; Li, H.; Zhang, X. Establishment of a miRNA-mRNA regulatory network in metastatic renal cell carcinoma and screening of potential therapeutic targets. *Tumour Biol* **2016**, doi:10.1007/s13277-016-5135-6.

21. Hubens, W.H.G.; Krauskopf, J.; Beckers, H.J.M.; Kleinjans, J.C.S.; Webers, C.A.B.; Gorgels, T. Small RNA Sequencing of Aqueous Humor and Plasma in Patients With Primary Open-Angle Glaucoma. *Invest Ophthalmol Vis Sci* **2021**, *62*, 24, doi:10.1167/iovs.62.7.24.
22. Liu, S.; Wang, H.; Mu, J.; Wang, H.; Peng, Y.; Li, Q.; Mao, D.; Guo, L. MiRNA-211 triggers an autophagy-dependent apoptosis in cervical cancer cells: regulation of Bcl-2. *Naunyn Schmiedebergs Arch Pharmacol* **2020**, *393*, 359-370, doi:10.1007/s00210-019-01720-4.
23. Zhong, Y.; Zhu, F.; Ding, Y. Differential microRNA expression profile in the plasma of preeclampsia and normal pregnancies. *Exp Ther Med* **2019**, *18*, 826-832, doi:10.3892/etm.2019.7637.
24. Shinawi, T.; Nasser, K.K.; Moradi, F.A.; Mujalli, A.; Albaqami, W.F.; Almukadi, H.S.; Elango, R.; Shaik, N.A.; Banaganapalli, B. A comparative mRNA- and miRNA transcriptomics reveals novel molecular signatures associated with metastatic prostate cancers. *Front Genet* **2022**, *13*, 1066118, doi:10.3389/fgene.2022.1066118.
25. Dandare, A.; Rabia, G.; Khan, M.J. In silico analysis of non-coding RNAs and putative target genes implicated in metabolic syndrome. *Comput Biol Med* **2021**, *130*, 104229, doi:10.1016/j.compbiomed.2021.104229.
26. Feng, C.; Li, Y.; Lin, Y.; Cao, X.; Li, D.; Zhang, H.; He, X. CircRNA-associated ceRNA network reveals ErbB and Hippo signaling pathways in hypopharyngeal cancer. *Int J Mol Med* **2019**, *43*, 127-142, doi:10.3892/ijmm.2018.3942.
27. Jing, L.; Jin, C.; Lu, Y.; Huo, P.; Zhou, L.; Wang, Y.; Tian, Y. Investigation of microRNA expression profiles associated with human alcoholic cardiomyopathy. *Cardiology* **2015**, *130*, 223-233, doi:10.1159/000370028.
28. Li, S.; Song, F.; Lei, X.; Li, J.; Li, F.; Tan, H. hsa_circ_0004018 suppresses the progression of liver fibrosis through regulating the hsa-miR-660-3p/TEP1 axis. *Aging (Albany NY)* **2020**, *12*, 11517-11529, doi:10.18632/aging.103257.
29. Liu, W.; Zhang, J.; Zou, C.; Xie, X.; Wang, Y.; Wang, B.; Zhao, Z.; Tu, J.; Wang, X.; Li, H.; et al. Microarray Expression Profile and Functional Analysis of Circular RNAs in Osteosarcoma. *Cell Physiol Biochem* **2017**, *43*, 969-985, doi:10.1159/000481650.
30. Zhao, X.; Dou, J.; Cao, J.; Wang, Y.; Gao, Q.; Zeng, Q.; Liu, W.; Liu, B.; Cui, Z.; Teng, L.; et al. Uncovering the potential differentially expressed miRNAs as diagnostic biomarkers for hepatocellular carcinoma based on machine learning in The Cancer Genome Atlas database. *Oncol Rep* **2020**, *43*, 1771-1784, doi:10.3892/or.2020.7551.
31. Yang, G.; Zhang, Y.; Yang, J. A Five-microRNA Signature as Prognostic Biomarker in Colorectal Cancer by Bioinformatics Analysis. *Front Oncol* **2019**, *9*, 1207, doi:10.3389/fonc.2019.01207.
32. Plata-Bello, J.; Farina-Jeronimo, H.; Betancor, I.; Salido, E. High Expression of FOXP2 Is Associated with Worse Prognosis in Glioblastoma. *World Neurosurg* **2021**, *150*, e253-e278, doi:10.1016/j.wneu.2021.02.132.
33. Wang, Y.; Li, Y.; Jia, D.; Zheng, J.; Wang, G. Correlation between single nucleotide polymorphisms in CXCR4 microRNA binding site and the susceptibility to knee osteoarthritis in Han Chinese population. *J Clin Lab Anal* **2021**, *35*, e23600, doi:10.1002/jcla.23600.
34. Ekmekci, C.G.; Coskunpinar, E.; Avci, H.; Farooqi, A.A.; Orhan, K.S.; Akbas, F. Integrative analysis of mRNA and microRNA expression profiles in laryngeal squamous cell carcinoma. *J Cell Biochem* **2019**, *120*, 3415-3422, doi:10.1002/jcb.27612.
35. Peng, Z.; Duan, Y.; Zhong, S.; Chen, J.; Li, J.; He, Z. RNA-seq analysis of extracellular vesicles from hyperphosphatemia-stimulated endothelial cells provides insight into the mechanism underlying vascular calcification. *BMC Nephrol* **2022**, *23*, 192, doi:10.1186/s12882-022-02823-6.
36. Chen, N.; Wan, G.; Zeng, X. Integrated Whole-Transcriptome Profiling and Bioinformatics Analysis of the Polypharmacological Effects of Ganoderic Acid Me in Colorectal Cancer Treatment. *Front Oncol* **2022**, *12*, 833375, doi:10.3389/fonc.2022.833375
37. Baker, M.A.; Davis, S.J.; Liu, P.; Pan, X.; Williams, A.M.; Iczkowski, K.A.; Gallagher, S.T.; Bishop, K.; Regner, K.R.; Liu, Y.; et al. Tissue-Specific MicroRNA Expression Patterns in Four Types of Kidney Disease. *J Am Soc Nephrol* **2017**, *28*, 2985-2992, doi:10.1681/ASN.2016121280.
38. Ju, L.; Han, M.; Li, X.; Zhao, C. MicroRNA Signature of Lung Adenocarcinoma with EGFR Exon 19 Deletion. *J Cancer* **2017**, *8*, 1311-1318, doi:10.7150/jca.17817.
39. Schoen, C.; Glennon, J.C.; Abghari, S.; Bloemen, M.; Aschrafi, A.; Carels, C.E.L.; Von den Hoff, J.W. Differential microRNA expression in cultured palatal fibroblasts from infants with cleft palate and controls. *Eur J Orthod* **2018**, *40*, 90-96, doi:10.1093/ejo/cjx034.
40. Pehserl, A.M.; Ress, A.L.; Stanzer, S.; Resel, M.; Karbiener, M.; Stadelmeyer, E.; Stiegelbauer, V.; Gerger, A.; Mayr, C.; Scheideler, M.; et al. Comprehensive Analysis of miRNome Alterations in Response to Sorafenib Treatment in Colorectal Cancer Cells. *Int J Mol Sci* **2016**, *17*, doi:10.3390/ijms17122011.

41. Wu, M.; Lu, L.; Dai, T.; Li, A.; Yu, Y.; Li, Y.; Xu, Z.; Chen, Y. Construction of a lncRNA-mediated ceRNA network and a genomic-clinicopathologic nomogram to predict survival for breast cancer patients. *Cancer Biomark* **2022**, doi:10.3233/CBM-210545.
42. Hu, J.; Zhou, S.; Guo, W. Construction of the coexpression network involved in the pathogenesis of thyroid eye disease via bioinformatics analysis. *Hum Genomics* **2022**, *16*, 38, doi:10.1186/s40246-022-00412-0.
43. Li, S.; Zhang, M.; Xu, F.; Wang, Y.; Leng, D. Detection significance of miR-3662, miR-146a, and miR-1290 in serum exosomes of breast cancer patients. *J Cancer Res Ther* **2021**, *17*, 749-755, doi:10.4103/jcrt.jcrt_280_21.
44. Liu, A.; Zhou, Y.; Zhao, T.; Tang, X.; Zhou, B.; Xu, J. MiRNA-3662 reverses the gemcitabine resistance in pancreatic cancer through regulating the tumor metabolism. *Cancer Chemother Pharmacol* **2021**, *88*, 343-357, doi:10.1007/s00280-021-04289-z.
45. Liu, A.; Xu, J. Circ_03955 promotes pancreatic cancer tumorigenesis and Warburg effect by targeting the miR-3662/HIF-1alpha axis. *Clin Transl Oncol* **2021**, *23*, 1905-1914, doi:10.1007/s12094-021-02599-5.
46. Byun, Y.J.; Piao, X.M.; Jeong, P.; Kang, H.W.; Seo, S.P.; Moon, S.K.; Lee, J.Y.; Choi, Y.H.; Lee, H.Y.; Kim, W.T.; et al. Urinary microRNA-1913 to microRNA-3659 expression ratio as a non-invasive diagnostic biomarker for prostate cancer. *Investig Clin Urol* **2021**, *62*, 340-348, doi:10.4111/icu.20200488.
47. Ye, J.; Xiao, X.; Han, Y.; Fan, D.; Zhu, Y.; Yang, L. MiR-3662 suppresses cell growth, invasion and glucose metabolism by targeting HK2 in hepatocellular carcinoma cells. *Neoplasma* **2020**, *67*, 773-781, doi:10.4149/neo_2020_190730N689.
48. Niveditha, D.; Jasoria, M.; Narayan, J.; Majumder, S.; Mukherjee, S.; Chowdhury, R.; Chowdhury, S. Common and Unique microRNAs in Multiple Carcinomas Regulate Similar Network of Pathways to Mediate Cancer Progression. *Sci Rep* **2020**, *10*, 2331, doi:10.1038/s41598-020-59142-9.
49. Chen, Z.; Zuo, X.; Zhang, Y.; Han, G.; Zhang, L.; Wu, J.; Wang, X. MiR-3662 suppresses hepatocellular carcinoma growth through inhibition of HIF-1alpha-mediated Warburg effect. *Cell Death Dis* **2018**, *9*, 549, doi:10.1038/s41419-018-0616-8.
50. Yasui, K.; Izumida, M.; Nakagawa, T.; Kubo, Y.; Hayashi, H.; Ito, T.; Ikeda, H.; Matsuyama, T. MicroRNA-3662 expression correlates with antiviral drug resistance in adult T-cell leukemia/lymphoma cells. *Biochem Biophys Res Commun* **2018**, *501*, 833-837, doi:10.1016/j.bbrc.2018.04.159.
51. Powrozek, T.; Kuznar-Kaminska, B.; Dziedzic, M.; Mlak, R.; Batura-Gabryel, H.; Sagan, D.; Krawczyk, P.; Milanowski, J.; Malecka-Massalska, T. The diagnostic role of plasma circulating precursors of miRNA-944 and miRNA-3662 for non-small cell lung cancer detection. *Pathol Res Pract* **2017**, *213*, 1384-1387, doi:10.1016/j.prp.2017.09.011.
52. Powrozek, T.; Mlak, R.; Dziedzic, M.; Malecka-Massalska, T.; Sagan, D. Analysis of primary-miRNA-3662 and its mature form may improve detection of the lung adenocarcinoma. *J Cancer Res Clin Oncol* **2017**, *143*, 1941-1946, doi:10.1007/s00432-017-2444-0.
53. Zhao, W.; Shen, W.W.; Cao, X.M.; Ding, W.Y.; Yan, L.P.; Gao, L.J.; Li, X.L.; Zhong, T.Y. Novel mechanism of miRNA-365-regulated trophoblast apoptosis in recurrent miscarriage. *J Cell Mol Med* **2017**, *21*, 2412-2425, doi:10.1111/jcmm.13163.
54. Maharry, S.E.; Walker, C.J.; Liyanarachchi, S.; Mehta, S.; Patel, M.; Bainazar, M.A.; Huang, X.; Lankenau, M.A.; Hoag, K.W.; Ranganathan, P.; et al. Dissection of the Major Hematopoietic Quantitative Trait Locus in Chromosome 6q23.3 Identifies miR-3662 as a Player in Hematopoiesis and Acute Myeloid Leukemia. *Cancer Discov* **2016**, *6*, 1036-1051, doi:10.1158/2159-8290.CD-16-0023.
55. Powrozek, T.; Krawczyk, P.; Kowalski, D.M.; Winiarczyk, K.; Olszyna-Serementa, M.; Milanowski, J. Plasma circulating microRNA-944 and microRNA-3662 as potential histologic type-specific early lung cancer biomarkers. *Transl Res* **2015**, *166*, 315-323, doi:10.1016/j.trsl.2015.05.009.
56. Li, Z.; Sun, Y.; He, M.; Liu, J. Differentially-expressed mRNAs, microRNAs and long noncoding RNAs in intervertebral disc degeneration identified by RNA-sequencing. *Bioengineered* **2021**, *12*, 1026-1039, doi:10.1080/21655979.2021.1899533.
57. Chen, W.; Tang, D.; Dai, Y.; Diao, H. Establishment of microRNA, transcript and protein regulatory networks in Alport syndrome induced pluripotent stem cells. *Mol Med Rep* **2019**, *19*, 238-250, doi:10.3892/mmr.2018.9672.
58. Lan, X.; Han, J.; Wang, B.; Sun, M. Integrated analysis of transcriptome profiling of lncRNAs and mRNAs in livers of type 2 diabetes mellitus. *Physiol Genomics* **2022**, *54*, 86-97, doi:10.1152/physiolgenomics.00105.2021.

59. Tarek, M.M.; Yahia, A.; El-Nakib, M.M.; Elhefnawi, M. Integrative assessment of CIP2A overexpression and mutational effects in human malignancies identifies possible deleterious variants. *Comput Biol Med* **2021**, *139*, 104986, doi:10.1016/j.compbiomed.2021.104986.
60. Morenikeji, O.B.; Adegbaju, M.S.; Okoh, O.S.; Babalola, A.E.; Grytsay, A.; Braimah, O.A.; Akinyemi, M.O.; Thomas, B.N. Deciphering inhibitory mechanism of coronavirus replication through host miRNAs-RNA-dependent RNA polymerase interactome. *Front Genet* **2022**, *13*, 973252, doi:10.3389/fgene.2022.973252.
61. Barbier, R.H.; McCrea, E.M.; Lee, K.Y.; Strope, J.D.; Risdon, E.N.; Price, D.K.; Chau, C.H.; Figg, W.D. Abiraterone induces SLCO1B3 expression in prostate cancer via microRNA-579-3p. *Sci Rep* **2021**, *11*, 10765, doi:10.1038/s41598-021-90143-4.
62. Hommers, L.G.; Richter, J.; Yang, Y.; Raab, A.; Baumann, C.; Lang, K.; Schiele, M.A.; Weber, H.; Wittmann, A.; Wolf, C.; et al. A functional genetic variation of SLC6A2 repressor hsa-miR-579-3p upregulates sympathetic noradrenergic processes of fear and anxiety. *Transl Psychiatry* **2018**, *8*, 226, doi:10.1038/s41398-018-0278-4.
63. Kasimanickam, V.; Kastelic, J. MicroRNA in sperm from Duroc, Landrace and Yorkshire boars. *Sci Rep* **2016**, *6*, 32954, doi:10.1038/srep32954.
64. Yilmaz, S.G.; Geyik, S.; Neyal, A.M.; Soko, N.D.; Bozkurt, H.; Dandara, C. Hypothesis: Do miRNAs Targeting the Leucine-Rich Repeat Kinase 2 Gene (LRRK2) Influence Parkinson's Disease Susceptibility? *OMICS* **2016**, *20*, 224-228, doi:10.1089/omi.2016.0040.
65. Wen, Q.; Verheijen, M.; Wittens, M.M.J.; Czurylo, J.; Engelborghs, S.; Hauser, D.; van Herwijnen, M.H.M.; Lundh, T.; Bergdahl, I.A.; Kyrtopoulos, S.A.; et al. Lead-exposure associated miRNAs in humans and Alzheimer's disease as potential biomarkers of the disease and disease processes. *Sci Rep* **2022**, *12*, 15966, doi:10.1038/s41598-022-20305-5.
66. Ulusan Bagci, O.; Caner, A. [miRNA Expression Profile in Ileocecal Adenocarcinoma Cells Infected with Cryptosporidium]. *Mikrobiyol Bul* **2022**, *56*, 449-465, doi:10.5578/mb.20229706.
67. Tripathi, S.K.; Mathaiyan, J.; Kayal, S.; Nachiappa Ganesh, R. Identification of Differentially Expressed Mirna by Next Generation Sequencing in Locally Advanced Breast Cancer Patients of South Indian Origin. *Asian Pac J Cancer Prev* **2022**, *23*, 2255-2261, doi:10.31557/APJCP.2022.23.7.2255.
68. Nguyen, D.D.N.; Zain, S.M.; Kamarulzaman, M.H.; Low, T.Y.; Chilian, W.M.; Pan, Y.; Ting, K.N.; Hamid, A.; Abdul Kadir, A.; Pung, Y.F. Intracellular and exosomal microRNAome profiling of human vascular smooth muscle cells during replicative senescence. *Am J Physiol Heart Circ Physiol* **2021**, *321*, H770-H783, doi:10.1152/ajpheart.00058.2021.
69. Jeong, S.; Kim, S.A.; Ahn, S.G. HOXC6-Mediated miR-188-5p Expression Induces Cell Migration through the Inhibition of the Tumor Suppressor FOXN2. *Int J Mol Sci* **2021**, *23*, doi:10.3390/ijms23010009.
